# 3D printed chip as platform to vascularize hiPSCs-derived kidney organoids

**DOI:** 10.1007/s10544-026-00829-7

**Published:** 2026-06-05

**Authors:** Gabriele Addario, Chiara Formica, Lorenzo Moroni, Carlos Mota

**Affiliations:** MERLN Institute for Technology-Inspired Regenerative Medicine, Complex Tissue Regeneration Department, Maastricht University, Maastricht, 6229 ER The Netherlands

**Keywords:** hiPSCs, Organoid, Kidney, 3D printing, Microvasculature

## Abstract

Human induced pluripotent stem cells (hiPSCs)-derived kidney organoids can resemble early stages of human kidney development, morphology and architecture. However, one of the main limitations of the organoids is the reduced vascularization, which limits differentiation and maturation. To increase the oxygen and nutrient supply, multiple vascularization strategies were proposed in literature, including organ-on-chip, hydrogels with angiogenetic cues, and co-culture with endothelial cells. In this work, we developed a three-dimensional (3D) printed chip by extruding sacrificial pluronic, in a fully automated and cost-effective way. By dissolving the pluronic, two circular cross-sectional channels, together with three separated central gel compartments, were created. Human umbilical vein endothelial cells (HUVECs) were seeded in the coated 3D printed chip, and after seven days kidney organoids were added in the central gel compartments, embedded in a partially digested decellularized extracellular matrix (ddECM) hydrogel, and co-cultured for five days under perfusion. At the end of the co-culture, capillary-like structures were formed towards the organoids both in the outer and central parts, colocalizing with LTL and PODXL positive stained areas. We were able to develop primitive capillary-like structures throughout the organoids, using an ad-hoc designed 3D printed chip. Our strategy provides new possibilities to investigate further organoid maturation, drug testing and disease modeling.

**Supplementary Information:**

The online version contains supplementary material available at 10.1007/s10544-026-00829-7.

## Introduction

Organoids are three-dimensional (3D) cellular structures derived from adult or pluripotent stem cells that self-organize and, upon maturation, resemble tissue morphology and functionality to a certain degree (Gupta et al. 2021; Yang et al. 2023). As described previously by our group (Formica et al. 2025), and others, several differentiation protocols were developed to generate induced pluripotent stem cells (iPSC) derived metanephric mesenchyme (MM) (Taguchi et al. 2014; Takasato et al. 2014, 2015; Freedman et al. 2015; Morizane et al. 2015; Morizane and Bonventre 2017), ureteric bud (UB) (Taguchi and Nishinakamura 2017; Mae et al. 2020; Uchimura et al. 2020; Zeng et al. 2021), and MM + UB organoids (Taguchi and Nishinakamura 2017; Uchimura et al. 2020; Tanigawa et al. 2022). These organoids have the capability to mimic early stages of the human kidney development, and can be used as disease models, however they do not recapitulate the complexity of the fully developed organ, as some cell populations are underrepresented, off-target cells are present, and maturation and vascularization are limited (Romero-Guevara et al. 2020; Dorison et al. 2022).

Kidneys are highly vascularized organs, receiving 20–25% of the cardiac output (Leckie et al. 2021). The renal tissue vascularization stems from vasculogenesis, which allows the formation of *de novo* blood vessels, and angiogenesis, where existing vessels branch and elongate (Gupta et al. 2021). Blood vessels are required to sustain long-term maturation, especially when the tissue size exceeds 100–200 μm, as above these distances oxygen and nutrient diffusion is often impaired (Place et al. 2017). Although a small portion of endothelial cell progenitor is normally formed during kidney organoids differentiation, most glomerular rudiments structures remain avascular (Nishinakamura 2019), and without organization to form blood vessels (Koning et al. 2020), denoting the difficulty in growing and maintaining a functional vasculature (Ryan et al. 2021). Furthermore, Ryan et al. suggested that the absence of blood flow may contribute to the regression of initially present endothelium in organoids derived from human embryonic stem cells (hESCs) and mouse embryonic nephrogenic zone cells (NZCs) (Ryan et al. 2021).

In order to enhance kidney vasculature, several strategies have been described in literature, such as the administration of vascular endothelial growth factor (VEGF) in vitro (Ryan et al. 2021), extracellular (ECM) coatings as decellularized ECM (dECM) and Matrigel^®^ (Lee et al. 2021; Kim et al. 2022), physiological 7% O_2_ hypoxia incubation (Schumacher et al. 2022), dynamic flow (Homan et al. 2019), or through methods using biofabrication technologies (Homan et al. 2019; Lee et al. 2021; Bas-Cristobal Menendez et al. 2022).

The kidney ECM, rich in collagens, proteoglycans, and glycoproteins, provides essential biochemical and mechanical cues that guide organogenesis and angiogenesis through integrin-mediated signaling (Valdoz et al. 2021; Khoshdel-Rad et al. 2022; Konoe and Morizane 2023). Reproducing this complex environment in vitro is challenging. Therefore, dECM and hydrogels derived from partially digested dECM (ddECM) have been developed to enhance the maturation and vascularization of human induced pluripotent stem cell (hiPSC)-derived kidney organoids (Poornejad et al. 2016).

Recent biofabrication approaches, including custom-built organ-on-chip systems and 3D bioprinting, have enabled the application of dynamic flow and ECM-like microenvironments, resulting in improved vascularization and glomerulus-like structures beyond the capabilities of static culture (Homan et al. 2019; Fransen et al. 2021; Lee et al. 2021; Aazmi et al. 2022; Bas-Cristobal Menendez et al. 2022; Kim et al. 2022). Nevertheless, current protocols still struggle to establish fully functional and interconnected vasculature, highlighting the need for new strategies to enhance vascularization strategies for kidney development in vitro.

In the current study, we aim to enhance the vascularization of the kidney (MM + UB) organoids, proposing a hybrid approach, including biofabrication, organoids, and hydrogels. Chips were 3D printed improving our first design (Addario et al. 2023), optimizing the medium for the co-culture of human umbilical vein endothelial cells (HUVECs) and hiPSCs-derived kidney organoids embedded in hydrogels. Metabolic activity and immunostaining studies were performed to access culture conditions and the vascular kidney structures within the developed 3D printed chip, respectively.

## Materials and methods

### HUVECs cell culture

HUVECs (Gibco, #10216773) were cultured in supplemented endothelial cell growth medium 2 (EGM-2, Bio-Connect, #C-22011), in cell culture treated T-flasks (VWR). Cells were seeded with a seeding density of 7.5 × 10^3^ cell/cm^2^ and cultured at 37 °C with 5% CO_2_ in an incubator. Upon reaching approximately 90% confluency, cells were washed with phosphate buffered saline (PBS, Sigma-Aldrich, #D8537), and trypsinized using 0.05% trypsin- ethylenediaminetetraacetic acid (trypsin-EDTA, 0.05%, Gibco, #11590626). HUVECs were used up to passage 6.

### hiPSCs culture and differentiation into MM + UB organoids

The hiPSCs (LUMC0031iCTRL08) were obtained from the Leiden University Medical Center (LUMC) hiPSC hotel core facility (Leiden, The Netherlands). LUMC0031iCTRL08 were generated from exfoliated renal epithelial cells with commercially available episomal plasmid. Cells were cultured in mTeSR™ plus (mTeSR plus STEMCELL Technologies, #05825) in treated 6 well plate (Thermo Fisher Scientific, #140685) coated with 1% v/v lactose dehydrogenase elevating virus (LDEV)-free human embryonic stem cell (ESC)-qualified (geltrex, Thermo Fischer, #12053569) in the incubator at 37 °C with 5% CO_2_. hiPSCs were passaged in colonies using a gentle cell dissociation reagent (STEMCELL Technologies, #07174) and used up to passage 27.

**Fig. 1:**
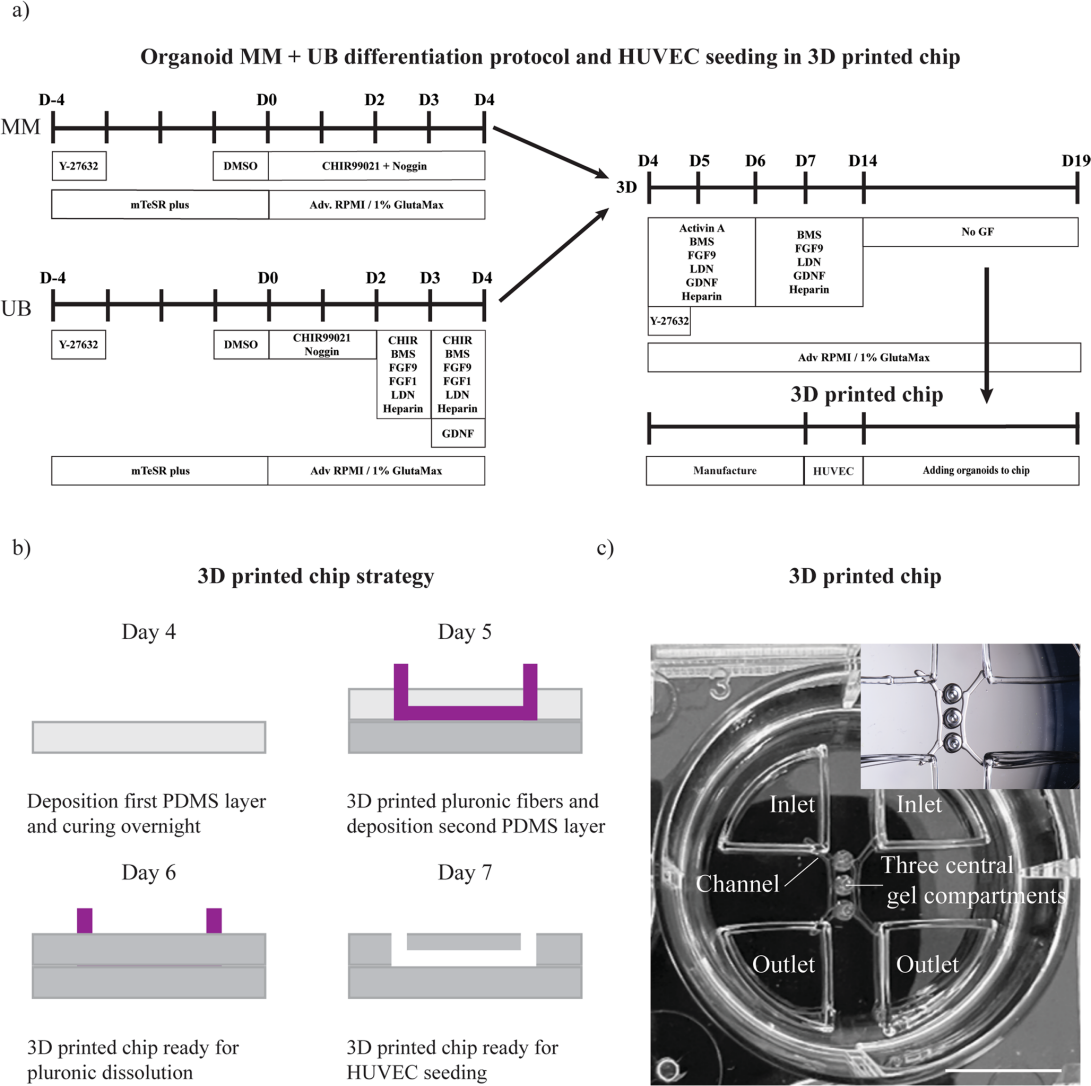
MM + UB organoid culture and 3D printed chip. (**a**) MM and UB were first cultured in 2D separately, and later co-cultured from Day 4 as 3D organoids. (**b**) 3D printing strategy for chip manufacturing and later HUVEC seeding: first a polydimethylsiloxane (PDMS) layer was deposited and cured overnight (Day 4 of organoid differentiation protocol), followed by the 3D printing of pluronic fibers on the top, later covered by a second layer of PDMS and cured overnight (Day 5), then the pluronic fibers were dissolved to create hollow channels and central gel compartments (Day 6), for HUVECs seeding (Day 7), and addition of organoids in the central gel compartments (Day 14). (**c**) Image of the 3D printed chip and insert image, highlighting the two inlets, two outlets and three central gel compartments between the two channels. Scale bar: 10 mm

MM and UB progenitors were first cultured separately in two-dimensional (2D), as previously described (Formica et al. 2025), and schematically reported in Fig. 1a, and then co-cultured from Day 4 (D4) as MM + UB 3D organoids. Once, the colonies reached 80% of confluency, they were detached into single cells using Accutase (STEMCELL Technologies, #07920), and seeded at a cell density of 1 × 10^4^ cells/cm^2^ on a 6-well tissue culture plates coated with 1% v/v geltrex, in mTeSR™ plus supplemented with 10 µM of ROCK inhibitor (Y-27632 dihydrochloride, Tocris, #1254) for one day. The following day, the medium was replaced with plain mTeSR™ plus medium. On Day − 1 (D-1) of the differentiation, the medium was removed from the cells and substituted with mTeSR™ plus supplemented with 1% v/v DMSO for 24 h. On Day 0 (D0), the medium was removed, and cells were washed with PBS. Advanced RPMI 1640 Medium (Adv RPMI, ThermoFisher Scientific, #11520446) supplemented with 1% v/v GlutaMax (ThermoFisher Scientific, #11574466), 8 µM of CHIR99021 (Tocris, #4423) and 5 ng/ml of recombinant human noggin (PeproTech, #120–10 C) was added, and cells were cultured up to Day 4 for MM. Whereas UB cells were cultured with the same medium composition of MM until Day 2 (D2). On Day 2 UB cells were cultured with Adv RPMI 1640 supplemented with 1% v/v GlutaMax, 1 µM CHIR99021, 0.1 µM BMS 493 (STEMCELL Technologies, #73972), 5 ng/ml FGF9 (STEMCELL Technologies, #78161.1), 100 ng/ml recombinant human FGF1 (PrepoTech, #100–17 A), 10 nM LDN-193,189 hydrochloride (Sigma-Aldrich, #SML0559-5MG), and 1 nM heparin sodium salt (Sigma-Aldrich, #H3393-10KU) between Days 2–3 (D2-D3). Thereafter, Days 3–4 (D3-4), UB cells were cultured in Adv RPMI 1640 and 1% v/v GlutaMax supplemented with 3 µM CHIR99021, 0.1 µM BMS 493, 5 ng/ml FGF9, 100 ng/ml FGF1, 10 nM LDN-193,189 hydrochloride, 1 nM heparin, and 2 ng/ml recombinant human Glial cell line-derived neurotrophic factor (GDNF, PrepoTech, #450 − 10).

On Day 4 of differentiation, the MM and the UB progenitor cells were detached and counted. A minimum of 20 × 10^4^ cells in a 1:1 ratio (MM: UB) were seeded in an ultra-low attachment, U-bottom 96-well tissue culture plate (Cellstar, #650 185), previously coated with 100 µl per well of anti-adherence rinsing solution (STEMCELL Technologies, #07010 ). Cells were centrifuged for 5 min at 200*g*, and Adv RPMI 1640 supplemented with 1% v/v GlutaMax,10 µM Y-27,632, 10 ng/ml activin A (premium grade, Miltenyi Biotec, #130-115-009), 0.1 µM BMS 493, 10 ng/ml FGF9, 10 nM LDN-193,189 hydrochloride, 2 ng/ml GDNF and 1 nM Heparin were added for 24 h. On Day 5 (D5), the medium was refreshed with the same mix of growth factors used on Day 4 without Y-27,632. On Day 6 (D6), the medium was replaced with Adv RPMI 1640 / 1% v/v GlutaMax supplemented with 0.1 µM BMS 493, 10 ng/ml FGF9, 10 nM LDN-193,189 hydrochloride, 2 ng/ml GDNF and 1 nM heparin, and refreshed every other day until Day 14 (D14) of differentiation. On Day 14, the organoids were cultured in Adv RPMI 1640 supplemented with 1% v/v GlutaMax, without any growth factors, for an additional 5 days, until Day 19 (D19). All the time points reported in the following sections are based on the differentiation protocol reported in Fig. 1a.

### Chip manufacturing through 3D printing of sacrificial template

Chips were manufactured adopting the same 3D printing of sacrificial pluronic strategy described before (Addario et al. 2023). Briefly, polydimethylsiloxane (PDMS) substrates were prepared using a Sylgard 184 kit (Dowsil, #101697) by mixing the silicone elastomer with the curing agent in a ratio of 10:1 in mass for 5 min. PDMS was degassed by centrifugation at 4500 rpm for 30 s. To create the bottom substrate, each well of a 6-non-treated well plate (VWR, #734–2777) was coated with 890 µl of PDMS. PDMS-coated surfaces were cured overnight at 40 ° C (Galaxy 14 S incubator, New Brunswick). A 40% w/v pluronic F-127 (Sigma-Aldrich, #P2443) solution was prepared by centrifuging the pluronic in autoclaved milliQ at 4 °C, for five minutes at 7000 rpm. The pluronic solution was loaded into a 10 ml cartridge with a 250 μm internal diameter nozzle (Nordson EFD, Inc) and connected to a N_2_ pressure-based system controlled by the bioprinter (BioScaffolder 3.1, GeSiM, Germany). Pluronic was 3D printed at 310 KPa, v = 60 mm/min, and offset of 500 μm. After pluronic extrusion, 750 µl PDMS was pipetted on top and cured overnight at 40 ° C. Afterwards, the pluronic fibers were dissolved by a washing step with PBS at 4 °C overnight, creating two hollow channels and three separated empty central gel compartments within the PDMS (Fig. 1b, c). The inlets and outlets were designed using Rhino version 6 software, while the g-code file was edited on the bioprinter software directly. These reservoirs replicated the curvature of the wall of the 6-well plates, accommodating 0.4 ml of medium in each of them. Images of acellular 3D printed chips were taken with a stereomicroscope (Motic SMZ-25).

### Metabolic activity

In view of the co-culture condition, the selection of appropriate medium composition was investigated through metabolic activity of HUVECs when exposed to different medium formulations. A resazurin-based cell metabolic activity assay (PrestoBlue™, Thermofisher Scientific) was used to evaluate the activity of HUVECs in different culturing media as: EGM-2, EGM-2 and Adv RPMI in 50:50 ratio in volume, and Adv RPMI (EGM-2 was supplemented, and Adv RPMI included 1% v/v GlutaMax). Cells were seeded at a cell density of 7.5 × 10^3^ cells/cm^2^ in a 96-treated well plate (ThermoFisher, #1021281), using EGM-2 medium. Once HUVECs formed a monolayer reaching confluency, they were cultured in the three tested media described above, and after three days, the resazurin-based solution was incubated for 1 h, at a dilution of 1:10 in culture medium. The measurements were made using a plate reader (CLARIOstar-BMG, Labtech), applying an excitation wavelength of 550 nm and an emission wavelength of 590 nm.

### ddECM hydrogel production

Kidneys were isolated from healthy pigs housed in the Central Animal Testing Facilities of Maastricht University, The Netherlands, through the tissue sharing program from ethically approved studies. Kidneys were washed in PBS, and the cortex tissue part was isolated, and further cut into small pieces as described before (Addario et al. 2024). Briefly, the tissue underwent a full week of incubations in multiple decellularization solutions as 0.1% w/v sodium dodecylsulfate (SDS, Sigma-Aldrich, #75746), 1% w/v SDS solution, 1% v/v triton X-100 (Merck, #T8787) solution, and 0.1% v/v peracetic acid 38–40% (Sigma-Aldrich, #1.07222.1000). Finally, the dECM was freeze-dried, and stored at -30 ° C until further use.

dECM was further partially digested (ddECM) in 0.1 M hydrochloridic acid (HCl, VWR, #310701.1000), adding 1 mg of pepsin from porcine gastric mucosa (Sigma-Aldrich, #P7125) for every 10 mg of dECM, at room temperature overnight on a stirring plate, reaching a final concentration of 2% w/v. The ddECM solution was equilibrated at neutral pH using 1 M sodium hydroxide (NaOH, VWR, #191373 M) and incubated at 37 ℃ to form a hydrogel.

### Proteomics analysis

The total protein concentration was quantified for the acellular ddECM, using a commercial kit (Pierce BCA protein assay kit, Thermo Fisher Scientific, #23227), following the manufacturer’s indications. 10 µg of the protein content was loaded into the wells of a precast polyacrylamide hydrogel (Mini-PROTEAN TGX, Stain-Free hydrogels, Biorad, #4568084), using as running buffer 10x Tris/Glycine/SDS (Biorad, #1610772), diluted in milliQ water to 1X. Hydrogel electrophoresis was set at 80 V for 5 min, followed by 1 h at 100 V. The pre-casted hydrogel was treated with Coomassie blue (Bio-Safe, Coomassie G-250 stain, Biorad, #161–0786), following the manufacturer’s protocol.

Protein bands were manually excised on a glass plate from the gel, and processed on a MassPREP digestion robot (Waters, Manchester UK). A solution of 50 mM ammonium bicarbonate (Sigma-Aldrich, #A6141) in 50% v/v acetonitrile (ACN, Biosolve, #012078) was used to remove the Coomassie blue. Cysteines were reduced with 10 mM dithiothreitol (Sigma-Aldrich, #10197777001) in 100 mM ammonium bicarbonate for 30 min followed by alkylation with 55 mM iodoacetamide (Sigma-Aldrich, #I1149) in 100 mM ammonium bicarbonate for 20 min. Spots were washed with 100 mM ammonium bicarbonate to remove excess reagents and were subsequently dehydrated with 100% ACN. Trypsin (6 ng/nl prepared in 50 mM ammonium bicarbonate) was added to the hydrogel plug and incubated at 37 ℃ for 5 h. The peptides were extracted with 1% v/v formic acid (FA, Biosolve, #069141) / 2% v/v ACN, and subsequently with 1% v/v formic acid / 50% v/v ACN. Peptide separation was performed on a Thermo Scientific (Dionex) Ultimate 3000 Rapid Separation Ultra-High-Performance Liquid Chromatography (UHPLC) system equipped with an Acclaim PepMap C18 analytical column (2 μm, 100Å, 75 μm x 150 mm). Peptide samples were first desalted on an online installed C18 trapping column. After desalting, peptides were separated on the analytical column with a 90-minute linear gradient from 5% to 35% ACN with 0.1% FA at 300 nL/min flow rate. The UHPLC system was coupled to a Q Exactive HF mass spectrometer (Thermo Scientific). Data was acquired in DIA mode with full ms scans between m/z 385–1,015 at a resolution of 60,000 and MS/MS scans at a resolution of 30,000. The LC-MS DIA data were analyzed with DIA-NN version 1.8.1. The SwissProt for *Sus scrofa* (pig) (SwissProt TaxID = 9823) and human database *Homo sapiens* (SwissProt TaxID = 9606) were used. The database search was performed with the following settings: enzyme was trypsin, a maximum of 2 missed cleavages, minimum peptide length of 7 amino acids, and a static modification of cysteine carbamidomethylation.

### 3D printed chip seeding and culture with HUVECs and organoids

Cell seeding was performed following the previously optimised protocol, with small adaptations (Addario et al. 2023). Briefly, after the dissolution of the 3D printed pluronic fibers with PBS at 4 °C, and the formation of the hollow channels and central gel compartments, the 3D printed chip was first incubated in 70% v/v ethanol (Boom, #84010059.5000) for 1 h at room temperature, followed by two PBS washes. Subsequently, channels and central gel compartment were coated with resolving gel buffer of 1.5 M trisaminomethane hydrochloride (Tris-HCl, Bio-Rad, #1610798) for 1 h at room temperature. Then the coating solution was added, that was a combination of a gelatin solution (EmbryoMax 0.1% gelatin, #ES-006-B), 0.5% v/v fibronectin bovine plasma (Sigma-Aldrich, #F1141), and 0.1% w/v dopamine hydrochloride solution (Sigma-Aldrich, #H8502), for one hour at 37 °C. After coating, the 3D printed chip was rinsed twice with PBS, and seeded with HUVECs at a cell density of 0.5 × 10^6^ cell/platform, using the corresponding medium.

In order to synchronize the HUVECs culture with the inclusion of organoids, 3D printed chips were prepared before Day 7 (D7) of organoid differentiation (Fig. 1a-b). On this day, HUVECs were seeded into the 3D printed chip and cultured in their native medium for additional 7 days to reach confluency in all the interior surfaces. On D14 MM + UB organoids were first mixed with 20 µl of 2% w/v ddECM (pH equilibrated), or 1% v/v geltrex, and then inserted in the central gel compartments. For the third central gel compartment, a gel-free condition (organoid only) was tested. Each gel compartment comprised one organoid embedded in the tested hydrogel, or in a gel-free condition. The co-culture of HUVECs and organoids in the 3D printed chip was kept in culture for extra five days, until D19 (Fig. 1a), in perfusion for three hours per day (Homan et al. 2019), using a rocking shaker (Grant bio PMR-30), at the speed of 5 oscillation/min in the Z-direction (Addario et al. 2024).

### Immunostaining and imaging

Organoids and 3D printed chips containing HUVECs with organoids were immunostained following the same procedure. The cellular samples were fixed with 4% v/v paraformaldehyde (PFA, 37% in H_2_O, 10–15% methanol, Sigma-Aldrich, #252549) for 30 min at room temperature, washed with PBS and permeabilized with 0.1% v/v Triton-X/PBS (permeabilization buffer) for 15 min on a rocking shaker at room temperature. Then the blocking buffer was added, comprising 10% v/v donkey or goat serum, 1% v/v Triton-X, 0.05% v/v Tween20 (VWR, #437082Q) in PBS, for 2 h on a rocking shaker at room temperature. The supernatant was removed, and the cellular samples were incubated with primary antibody diluted in 1.5% w/v bovine serum albumin (BSA, VWR, #421501J) in PBS overnight on a rocking shaker at 4 °C to their respective working dilutions: LTL-Fluorescin (1:100, Brunschwig Chemie#FL-1321), mouse anti-CD31 (1:500, Abcam, #ab24590), rabbit anti-GATA3 (1:500, CST, #5852T), and goat anti-Podocalyxin (1:100, R&D Systems, #AF1658). The day after, the supernatant was discarded, and three washes were performed with permeabilization buffer for 1 h each. Subsequently, the cellular samples were incubated with secondary antibody diluted to their respective working dilutions, overnight on a rocking shaker at 4 °C: donkey α-goat Alexa Fluor 568 (1:1000, ThermoFisher, #10463972), goat α-rabbit 647 (1:1000, ThermoFisher, #10543623), goat α-mouse Alexa Fluor 647 (1:1000, ThermoFisher Scientific, #A-21240). The day after, 3 washes with permeabilization buffer were performed for periods of 1 h each. Then, the organoids or 3D printed chips were incubated with 4′,6-diamidino-2-phenylindole (1:100, DAPI, Sigma-Aldrich, #32670-5MG-F) for 15 min at room temperature, washed with permeabilization buffer for 15 min. All the washes were performed at room temperature on a rocking shaker. For the 2D HUVEC culture, the same fixation and immunostaining protocol was performed, however the fixation lasted 10 min, and the secondary antibody was incubated for one hour at room temperature.

Finally, the samples were kept in PBS and imaged with a fluorescence microscope (automated inverted Nikon Ti-E microscope, equipped with a LumencorSpectra X light source, Photometrics Prime 95B sCMOS camera, an MCL NANO Z500‐N TI z‐stage), and confocal microscope (Leica TCS SP8 STED). Z stack images were deconvoluted using NIS Elements 5.21.01, the multiple channels merged with Fiji ImageJ 1.53 K, and compiled into the presented figures using Adobe Illustrator 2020. CD31 immunofluorescence images were quantified using Fiji. RGB TIFF images were split into individual channels, the CD31 channel was converted to 8-bit grayscale, and a uniform threshold was applied. Vascularization was quantified as CD31-positive area fraction (% area of total image).

### Gene expression study

Gene expression was evaluated for multiple vascularization genes, as shown in Table 1, following a previous publication from our group (Addario et al. 2024). Pellet pestles (Fisher Scientific, #11 815 125) were used to first break down the organoids with or without the ddECM hydrogel, then incubated in 2 mg/ml collagenase from clostridium hystolyticum (Sigma–Aldrich, #C5894) for 5 min using the thermomixer (Eppendorf, ThermoMixer C) at 37 °C, 500 rpm, and finally adding TRIzol™ (Fisher Scientific, #15 596 018). The total RNA was extracted using the chloroform/isopropanol method. The RNA was reverse transcribed using iScript cDNA synthesis kit, according to the manufacturer protocol. Quantitative PCR (qPCR) was carried out using Biorad-CFX96 with IQ SYBR green mix (Biorad, Cat #1 708 886). Gene expression was normalized to the housekeeping gene *HPRT1*, and the fold change was calculated using the 2^−ΔΔCt^ method.

**Table 1:** qPCR primers’ sequences. List of primers used and their sequence

Primer list	Sequence F	Sequence *R*
HPRT1	TTGTTGTAGGATATGCCCTTGAC	GGACTCCAGATGTTTCCAAACTC
KRT8	CTCCACTTGGTCTCCAGCAT	GGAGCAGATCAAGACCCTCA
VIM2	GCCGAAAACACCCTGCAATC	TCCTGGATTTCCTCTTCGTGG
PODXL	AACCCGGCCCAAGATAAGTG	TTGGCAGGGAGCTTAGTGTG
VEGF A	AGGGCAGAATCATCACGAAGT	AGGGTCTCGATTGGATGGCA

### Statistical analysis

Statistical analysis was performed using GraphPad Prism8 (version 8.2.0) software, implementing a one-way ANOVA or a t-test. A p value smaller than 0.05 was considered statistically significant (*p < 0.05, **p < 0.01, ***p < 0.005, ****p < 0.0001 and ns for *p* > 0.05).

## Results

### Metabolic activity of HUVECs in different culture media

**Fig. 2:**
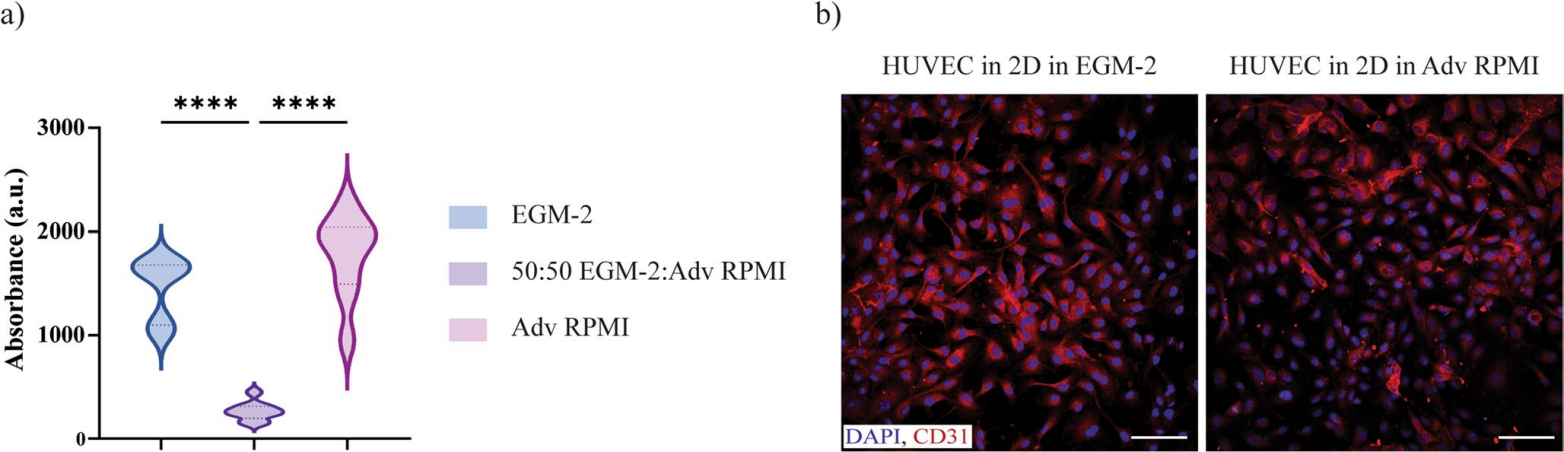
Optimizations of the medium composition for the HUVEC cultured in the chip. HUVECs were cultured in 2D in three medium formulations, namely EGM-2, Adv RPMI and 50:50 EGM-2:Adv-RPMI, and (**a**) the metabolic activity (*n* = 9) was measured first, together with (**b**) immunostaining with DAPI (in blue), and CD31 (in red), for HUVECs cultured in EGM-2 and Adv RPMI, as produced the highest metabolic activities measured. Scale bars: 100 μm

To co-culture the endothelial cells with the organoids from D14 onwards in the 3D printed chip, a screening for the selection of the suited medium was required. Considering that both HUVECs and organoids required different culture media, we investigated the effect of culturing the endothelial cells with the mixture of culture media in a 50:50 ratio, or with Adv RPMI (organoid medium after D14), and compared with EGM-2 (endothelial medium recommended by the HUVECs provider). HUVECs’ metabolic activity was measured with PrestoBlue™ when cultured with the different media. Adv RPMI medium yielded the highest metabolic activity and resulted similar to HUVECs’ native medium, EGM-2, while the 50:50 mixture resulted in the lowest metabolic activity (Fig. 2a). Based on these results, HUVECs were cultured in 2D both in EGM-2 and Adv RPMI, and stained for the endothelial marker CD31 (Fig. 2b). Images showed that HUVECs expressed CD31 in both conditions, confirming the endothelial cells can be cultured in Adv RPMI medium, maintaining their endothelial phenotype.

### Co-culture of HUVECs and organoids: testing gel formulations and culture media

**Fig. 3:**
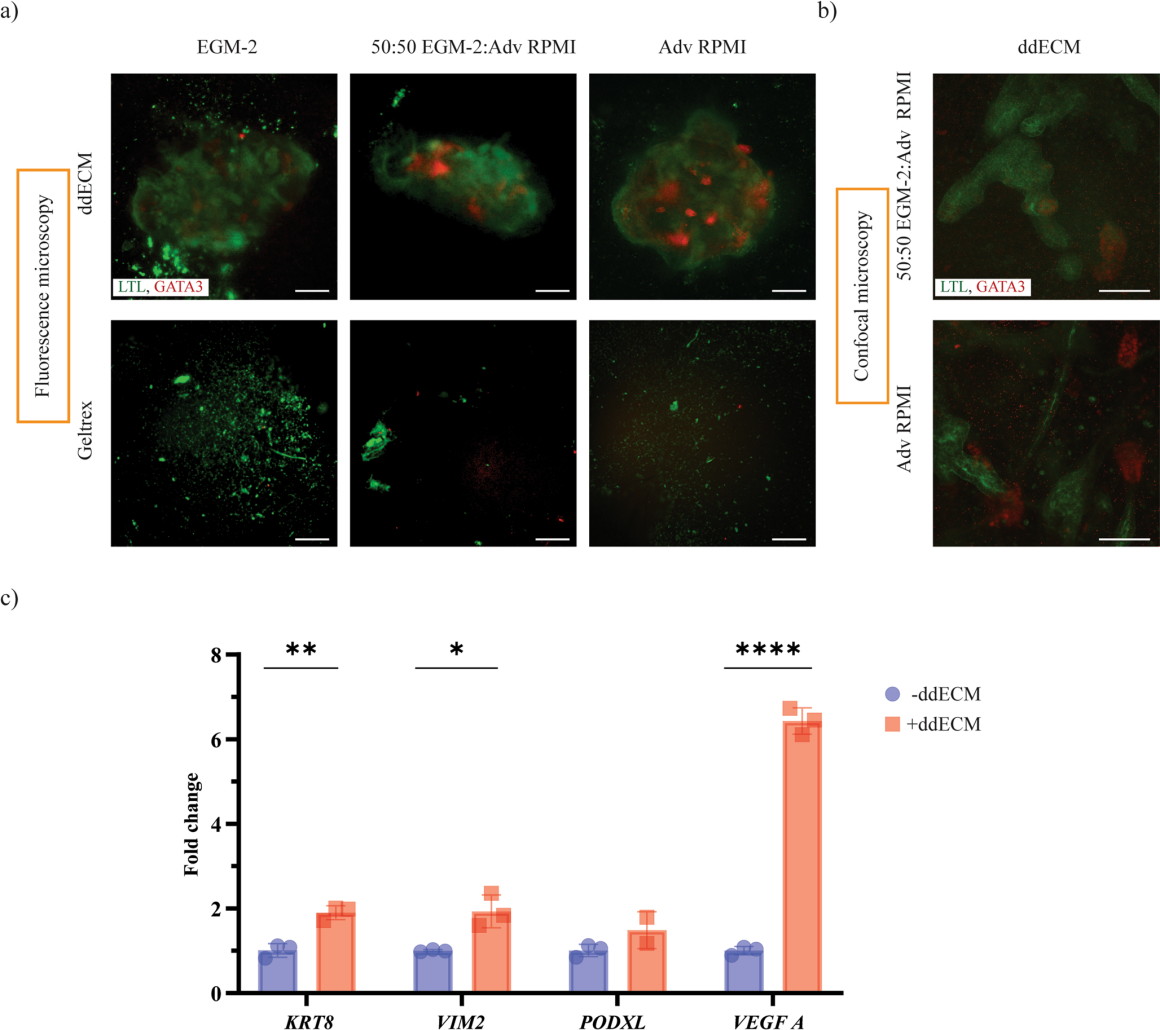
Medium and hydrogel optimization for co-culture of HUVECs and MM+UB organoids in the 3D printed chips. kidney organoids were cultured in two different gel formulations of 2% w/v ddECM and 1% w/v geltrex, and in three different medium formulations, namely EGM-2, 50:50 EGM-2: Adv RPMI, and Adv RPMI, within 3D printed chips. Immunostaining with (**a**) fluorescence microscopy imaging was performed, and the best conditions were further imaged with (**b**) confocal microscope, for LTL (in green) and GATA3 (in red). (**c**) The gene expression analysis was conducting to understand if the ddECM hydrogel could enhance the vascularization of the organoid on Day 19, *N*=3, *n*=1. Scale bars: 200 µm for **a**), and 100 µm for **b**)

From Day 4, MM and UB were co-cultured using a specific cocktail of GFs (Fig. 1a) as previously reported (Formica et al. 2025). HUVECs were seeded in the 3D printed chips on Day 7 and cultured in EGM-2 medium for one week. On Day 14, kidney organoids were embedded in two different gel formulations, or in a gel-free condition, and added into the 3D printed chip: in the top gel compartment the organoid was embedded in ddECM, in the central gel compartment in a gel-free condition, while in the bottom gel compartment in geltrex. The organoid in the gel-free condition was not stable within the 3D printed chip compartment, and it was washed away within a couple of days. Whereas the hydrogels were able to constrain the organoids within the gel compartments of the 3D printed chip (Figure S1). We also evaluated the effect of the three tested medium compositions (EGM-2, 50:50 EGM-2:Adv RPMI, and Adv RPMI) on the morphology of the organoids embedded into ddECM or geltrex hydrogel formulations cultured from Day 14 to Day 19. The organoids embedded in geltrex lost their 3D structure over time, regardless of the culture medium formulation used (Figure 3a). However, the organoids embedded in the ddECM were capable of maintaining their 3D architecture (Figure 3a). In this condition, the organoids resulted positively stained for LTL and GATA3 for all the tested culture media (Figure 3a). More visible proximal and distal tubules, resembling nephron’s tubular structures could be seen in 50:50 and Adv RPMI medium compositions. Therefore, the conditions 50:50 EGM-2:Adv RPMI and Adv RPMI were further imaged with confocal microscopy to acquire a detailed view of these structures throughout the volume of the organoid embedded in ddECM (Figure 3b). The immunostaining showed the presence of proximal and distal tubule structures stained by LTL and GATA3 respectively, demonstrating a continuous connection between these kidney structures. Considering these results together with the metabolic activity presented in Figure 2a, we decided to use Adv RPMI to perform the co-culture of the ddECM embedded organoids within a pre-endothelialized chip.

### Vascularization of kidney organoids within the 3D printed chip

Proteomic analysis of the ddECM identified several proteins associated with endothelial cell adhesion, migration, and angiogenesis, including fibronectin, vimentin, integrin β-1, laminin subunits β-1 and β-2, and moesin (Table S1). To evaluate the ability of the ddECM to support endothelial migration, a ddECM gel was casted above a 2D monolayer of HUVECs. After two days, HUVECs migrated and fully colonized the 3D ddECM gel, as observed in the supplementary videos (Video S1, S2). Next, we investigated the effect of embedding kidney organoids in the ddECM on gene expression (Fig. 3c). A statistically significant upregulation of *KRT8*, *VIM*, and *VEGFA* was detected compared with gel-free organoids, whereas *PODXL* expression did not show significant changes. The increased expression of *VEGFA* suggests the development of vascular-like structures. At the same time, the expression of genes *KRT8*, *VIM*, and *PODXL* indicate maintenance of epithelial, mesenchymal, and podocyte populations, respectively.

.  

**Fig. 4:**
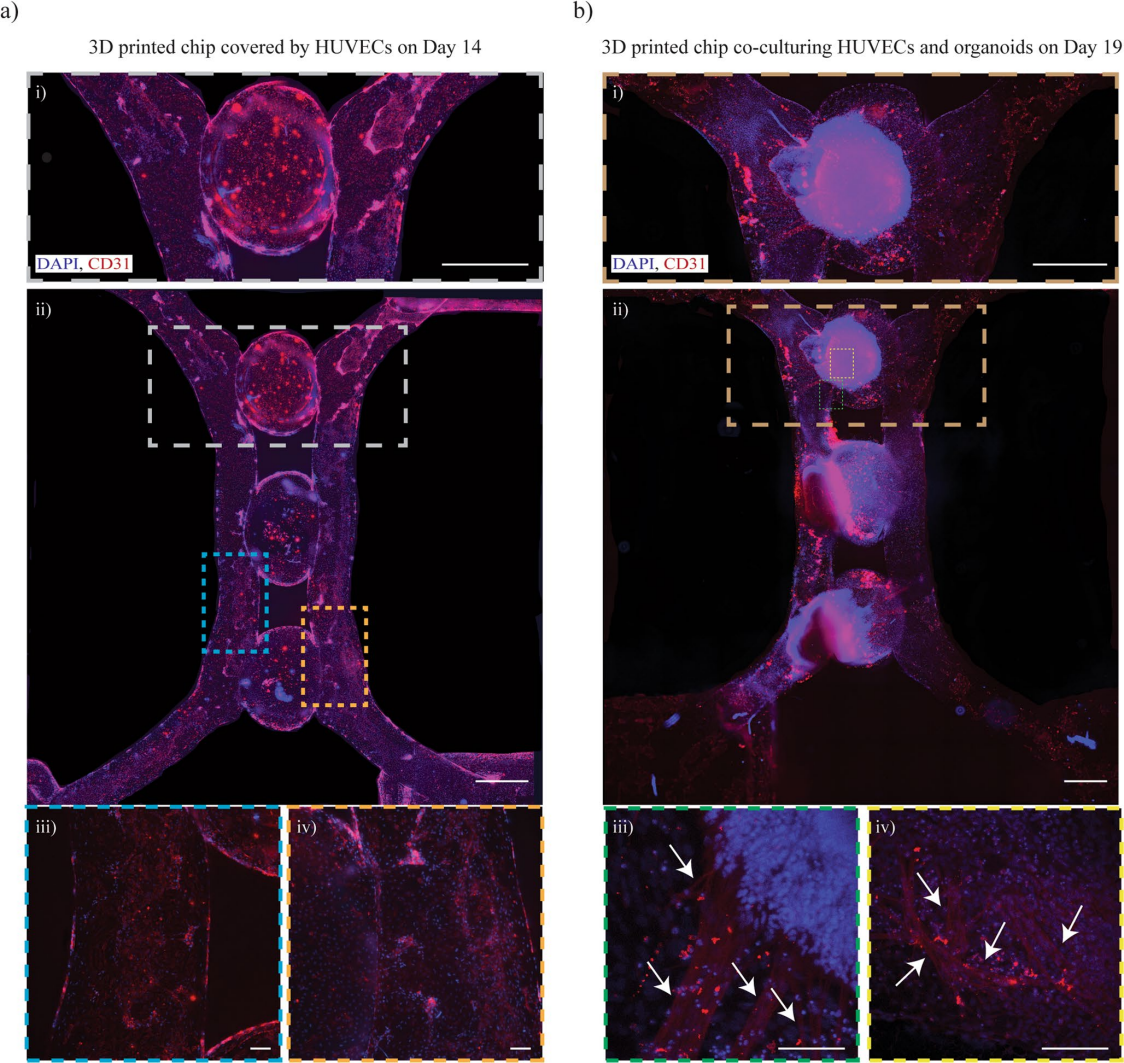
Branching formation for kidney organoids when co-cultured with HUVECs in the 3D printed chips. The 3D printed chip were first seeded with HUVEC cultured for seven days showing a full coverage of the inner surfaces of the 3D printed chip (Day 7–14), as shown in (**a**), together with detailed images i-iv). Then, embedded organoids in ddECM were added in the 3D printed chip, changing the medium from EGM-2 to Adv RPMI, showing the formation of branching, as shown in (**b**) (Day 19), together with zoom in images i-iv), highlighted by the white arrows. Scale bars: 1000 μm for i), and ii), whereas 100 μm for zoom in images for iii), and iv), both in **a**) and **b**). DAPI staining is shown in blue and CD31 in red

**Fig. 5:**
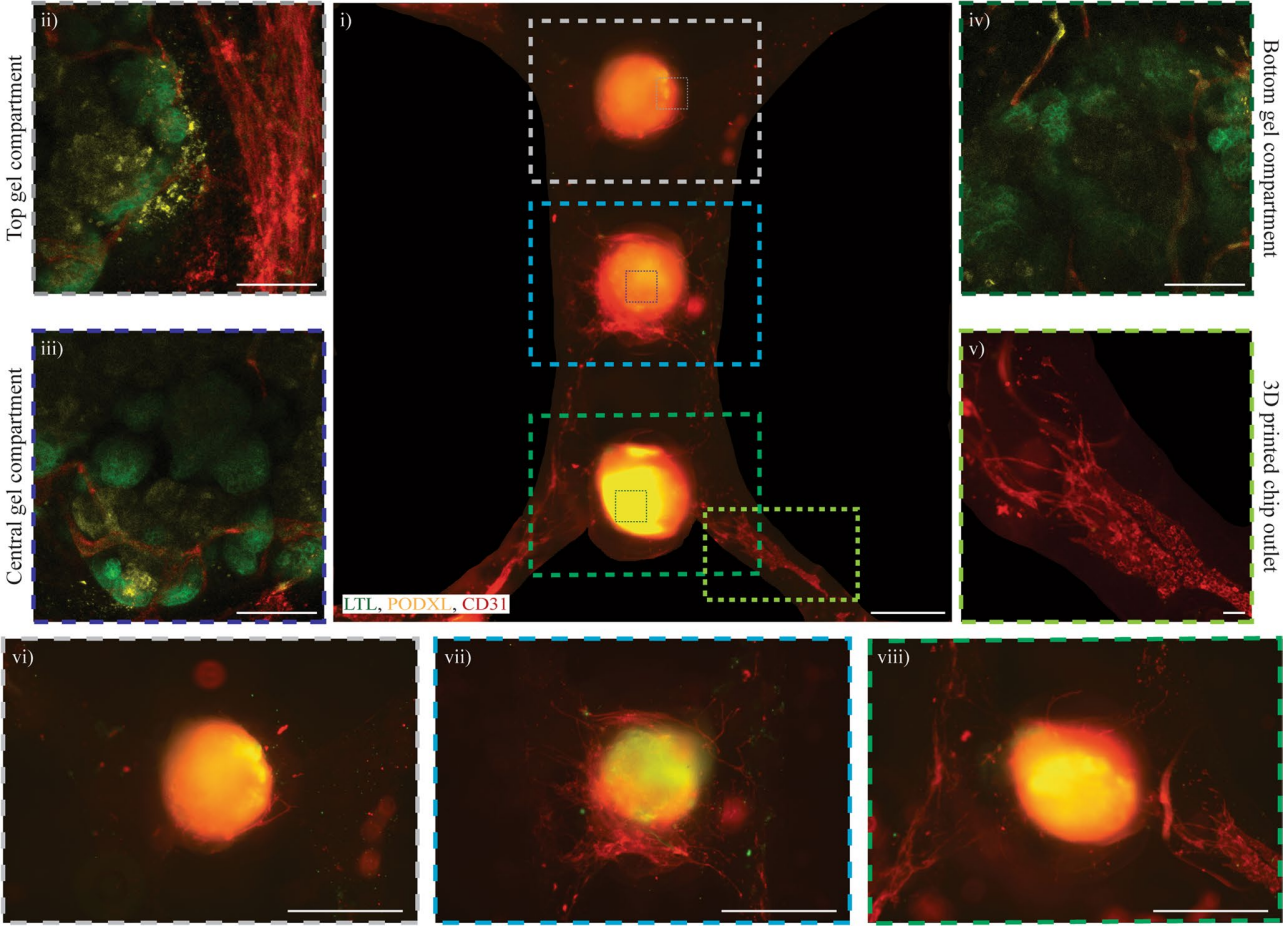
Vascularization of organoids showing nephron-like structures. The formation of branching throughout the three organoids was characterized by immunostaining of pre-endothelialized chips with HUVECs combined with embedded ddECM organoids, and cultured up to day 19 as reported in i). Moreover, the three organoids in the top ii), central iii), and bottom gel compartments iv) showed proximal tubule-like and glomerulus-like structures stained by LTL (in green) and PODXL (in yellow) respectively, with the formation of capillary-like structures (stained in red) within the organoids. v) A rearrangement of the monolayer of HUVECs was also observed with newly formed branches in the macro-channels, stained by CD31 (in red), further investigated by a closer look at the ddECM embedded organoids in the vi) top gel compartment, vii) central gel compartment, viii) and bottom gel compartment. Scale bars: 1000 μm for i) and 100 μm for ii-viii)

To integrate kidney organoids within pre-vascularized chips, HUVECs were cultured in the microfluidic chip for seven days (Day 7–Day 14 of the kidney differentiation protocol). During this period, HUVECs formed a confluent monolayer along the inner surfaces of the chip (Fig. 4a i–iv), covering 79.36% of the total chip area positive for CD31. On Day 14, one organoid embedded in the ddECM hydrogel was added in each of the three central gel compartments of the HUVEC-seeded 3D printed chip. At this stage, the culture medium was switched from EGM-2 to Adv RPMI. After five days in culture (Day 19), remodeling of the HUVEC layer and the formation of branching structures were observed (Fig. 5b i–iv; Video S3, S4), with CD31-positive cells covering 72.90% of the total chip area. Primitive capillary-like structures were visible both at the periphery and within the interior of the organoids, as indicated in Fig. 4b iii–iv (white arrows) and in the supplementary videos (Video S3, S4).

Two control conditions were performed. In the first, Day-14 kidney organoids were embedded in ddECM and cultured in U-well plates: no branching structures were observed after five days (Figure S2). In the second, organoid-free HUVEC-seeded chips were maintained in Adv RPMI medium until Day 19: no branching formation was detected, and the CD31-positive coverage was reduced to 39.01% of the total chip area (Figure S3).

To assess the localization of the newly formed vasculature towards the organoids previously described, further characterization of key kidney organoid markers was performed with immunostaining (Fig. 5). On Day 19, the organoids showed positively stained areas for LTL, PODXL and CD31 in all the three organoids placed in the central gel compartments, denoting the consistency of the vascularization, with 44,04% CD31-total covered chip area (Fig. 5ii-iv). We further processed the images of the central gel compartments, creating videos showing the formation of capillary-like structures stained with CD31, that interacted with the proximal tubule-like and glomerulus-like areas, stained by LTL and PODXL respectively (Video S5-S7).

In line with the previous results shown in Fig. 4b, the HUVECs seeded in the channel of the 3D printed chip changed morphology, from a monolayer to capillary-like structures as approaching the organoids in the central gel compartments (Fig. 5v). These results were further proved by a closer analysis of the three central gel compartments, detecting the formation of branching throughout all the three organoids (Fig. 5vi-viii).

## Discussion

Kidney diseases affect over 750 million people worldwide, posing a significant social and economic burden (Wu et al. 2022). However, there is still a lack of humanized 3D in vitro models that can serve as reliable platforms for drug testing and therapeutic development. 2D cell cultures are still used nowadays, but these screening systems present challenges to replicate the kidney cellular population and architecture (Schutgens et al. 2016). On the other hand, kidney organoids can provide new insights into kidney organogenesis, emulate pathological conditions, and provide a new platform for drug testing and therapy development (Koning et al. 2020; Takasato and Wymeersch 2020; Shimizu et al. 2021). Although the research on kidney organoids has significantly advanced over the last years, with multiple protocols for the differentiation of hiPSCs becoming available (Koning et al. 2020), more work is required to improve the maturation of these cellular organ rudiments closer to that of the fully developed organ. One of the main limitations of kidney organoids is the reduced vascularization, affecting stem cell differentiation (Rymer et al. 2014), and maturation (Tekguc et al. 2022).

In the present work, we proposed a fully automated process to produce unexpensive 3D printed chips, with pluronic as sacrificial material. 3D printing allowed to manufacture templates, which are then encapsulated in PDMS resulting in 3D printed chips. The produced channels have a circular cross-section, mimicking the native capillaries, a feature that cannot be easily achieved using other biofabrication strategies, such as stereolithography. Menéndez et al. adopted a commercially available chip with rectangular cross-sectioned channels seeded with fluorescent HUVECs for two days, followed by the addition of hiPSCs-derived kidney organoid, showing that HUVECs were able to invade the organoid after nine days in co-culture, under perfusion (Bas-Cristobal Menendez et al. 2022). In this model, the organoids were placed in a culturing chamber on the top of three perfusable channels, not directly in contact with the seeded HUVECs into the channels. This configuration might explain the reduced vascularization of the organoid limited to the outer edge, which contrasts with our findings where capillary-like structures were present both around and within the organoids. Furthermore, the HUVECs were only cultured for two days, showing a reduced monolayer formation. The medium composition for the co-culture was different from the one reported herein: mixed medium was adopted compared to only Adv RPMI. This might be explained by the different HUVEC and hiPSCs lines adopted amongst the models.

Lee et al. proposed an in-house manufactured chip, produced by a 3D printed mold later used for PDMS casting, presenting microwells coated with VEGF and Matrigel^®^ for culturing multiple organoids in the same system, under flow condition (Lee et al. 2021). The combination of flow and coating proved to enhance endothelial markers such as PECAM1. This approach resembled our strategy in providing a platform to test and vascularize multiple organoids in the same system, using an in-house approach. However, the latter published model presented a different geometry, showing a chip formed by microwells for the simultaneous culture of multiple organoids, without the inclusion of channel-like structures. Whereas in our 3D printed chip we manufactured two channels, and three central gel compartments that allowed to test multiple gel-formulations and gel-free condition in the same 3D printed chip, near the manufactured channels.

Finally, Homan et al. produced a perfusable platform made of 3D printed PDMS gaskets (Homan et al. 2019). This platform allowed to test different conditions, where hPSCs differentiated in MM and SIX2^+^ nephron progenitor to form organoids, that were co-cultured with HUVECs, human glomerular microvascular endothelial cells (GMECs), or human neonatal dermal fibroblasts (HNDFs). Multiple culture media were tested, namely Adv RPMI, EGM-2, or the combination, with the addition of fetal bovine serum (FBS), and all showed no increased of vascularization. Furthermore, the model included the addition of the above-mentioned primary cells on Day 8 of the differentiation, while, in our study, we first concluded the differentiation up until Day 14, and later co-cultured the organoid with the HUVECs pre-seeded in the 3D printed chip.

Another important difference between our work and some of the previously described models (Lee et al. 2021; Bas-Cristobal Menendez et al. 2022) is the 3D embedding of the organoids in hydrogels. It was described that hydrogels can boost the maturation and differentiation of kidney organoids, as normally organoids are cultured on static plastic surfaces, whereas a soft, dynamic and mechanically compliant material would be ideal (van Sprang et al. 2022). In our model, the organoids were embedded in ddECM, while in a previously reported study a ddECM 2D coating with the addition of VEGF showed to strongly increase the vascularization of hiPSCs-derived kidney organoids, and the formation of glomerulus-like structures (Kim et al. 2022). However, in our work we were not able to prove the presence of the VEGF in the ddECM formulation, but several other pro-angiogenic proteins were detected. We determined fibronectin, an essential ECM protein for blood vessel morphogenesis (Sophie Astrof 2009), and angiogenesis (Christie J. Avraamides 2008), together with its receptor integrin β-1, both of which are relevant for vascular development (Christie J. Avraamides 2008). Similarly, vimentin was recognized as a pro-angiogenic factor that emulates VEGF activity, facilitating both cellular migration and capillary-like tube formation (Judy R. van Beijnum 2022, Sepideh Parvanian 2023). In parallel, moesin has been reported as a critical mediator of angiogenesis, primarily through its regulation of the RhoA/ROCK signaling pathway (Qian Wang 2016). These results are in line with the study of Kim et al. (Kim et al. 2022), in which the inhibition of VEGF did not restrict vascular growth, suggesting that ddECM provides a complex mixture of growth factors, that extends beyond VEGF alone to support organoid maturation (Kim et al. 2022; Konoe and Morizane 2023). We hypothesized that the observed branching may be attributed to the presence of multiple pro-angiogenic proteins within the ddECM. On the other hand, the gene expression results collectively demonstrated that the kidney organoids exhibited key features of nephron-like units and associated vascular components, further enhanced by the encapsulation with ddECM. This follows previously published literature (Kim et al. 2022; Konoe and Morizane 2023), reporting ECM supports mechanically and biochemically cell proliferation and differentiation.

The 3D-printed chip developed in this study represents a significant advancement over our previous platform, where primary cells were maintained under static conditions (Addario et al. 2023). Dynamic flow has been widely reported to enhance vascularization in 3D in vitro models (Michael Lovett 2009), whereas static culture conditions support little or no vascular network formation for kidney organoids (Homan et al. 2019; Lee et al. 2021; Raykhel et al. 2024). By implementing this approach, we observed direct interactions between CD31⁺ capillary-like networks and PODXL⁺ glomerulus-like regions. Establishing this connection is crucial for the maturation of kidney organoids, as current protocols have largely failed to generate functional vasculature with integration into the glomerular compartment, a key step toward replicating glomerular filtration (Raykhel et al. 2024).

Finally, the central gel compartments allowed us to test multiple hydrogel formulations for organoid embedding, within the same 3D printed chip. It was also possible to obtain consistent replicates in the same platform, when the hydrogel formulation and culture medium were optimized, features that were not reported in the previously described studies (Homan et al. 2019; Lee et al. 2021; Bas-Cristobal Menendez et al. 2022).

## Conclusion

In this work, we developed a platform to increase the vascularization of hiPSCs organoids, embedded in ddECM and co-cultured in a pre-vascularized chip with HUVECs. An ad-hoc chip was manufactured through PDMS encapsulation of a 3D printed template. Early vascularization was shown, together with the formation of capillary-like structures, both in the outer and inner parts of the organoids. Furthermore, the co-cultured organoids showed nephron-like structures, both proximal tubule and glomerulus, as shown by the positively stained areas by LTL and PODXL respectively. In conclusion, the developed model for the vascularization of kidney organoids might allow further maturation. Mature and functional kidney organoids are essential for future implementation in drug testing and disease modelling, such as chronic kidney disease (CKD) and kidney fibrosis.

## Supplementary information

Below is the link to the electronic supplementary material.

Supplementary File 1 (avi 938 KB)

Supplementary File 2 (avi 2.18 MB)

Supplementary File 3 (avi 9.46 MB)

Supplementary File 4 (avi 6.69 MB)

Supplementary File 5 (avi 2.91 MB)

Supplementary File 6 (avi 1.71 MB)

Supplementary File 7 (avi 1.28 MB)

Supplementary File 8 (DOCX 3.38 MB)

## Data Availability

All data supporting the findings of this study are available within the paper and its Supplementary Information.
