# Supplementary material for: 3D printed chip as platform to vascularize hiPSCs-derived kidney organoids
Source: Biomed Microdevices. 2026 Jun 5;28(2):49. doi: 10.1007/s10544-026-00829-7 (PMC13241417; doi:10.1007/s10544-026-00829-7)
Supplement: Supplementary file 8 — (DOCX 3.38 MB) [file 10544_2026_829_MOESM8_ESM.docx]

# Supporting information

3D printed chip as platform to vascularize hiPSCs-derived kidney organoids

Gabriele Addario, Chiara Formica, Lorenzo Moroni, Carlos Mota*


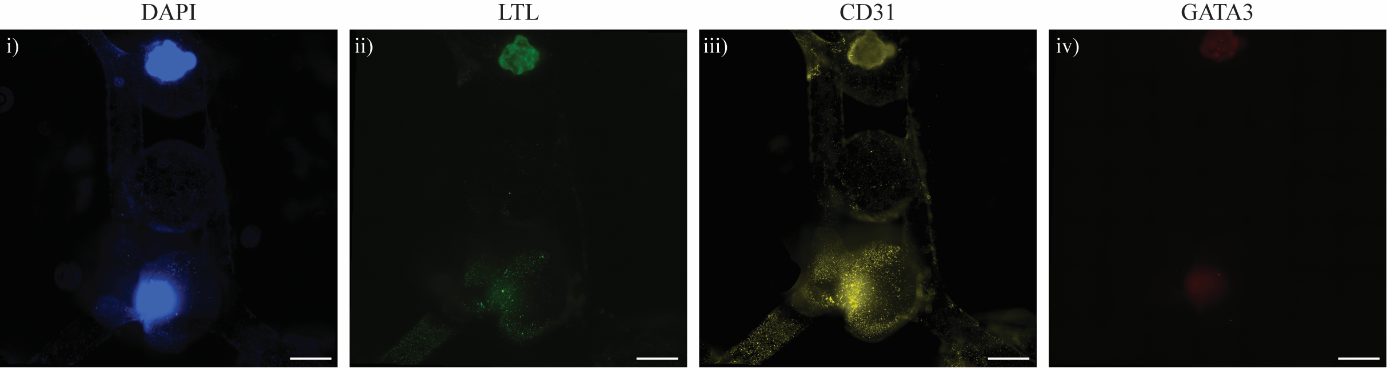


Figure S1: **Representative image of an overall chip testing multiple gel and gel-free conditions.** Multiple conditions were tested, as in the top gel compartment the organoid was embedded in ddECM, in the central gel compartment in a gel-free condition, while in the bottom gel compartment in geltrex. Results showed that the organoid in the central gel-free condition was washed away, as reported by immunostaining on Day 19, for DAPI in blue (i), LTL in green (ii), CD31 in yellow (iii) and GATA3 in red (iv). Scale bar: 1000 µm.


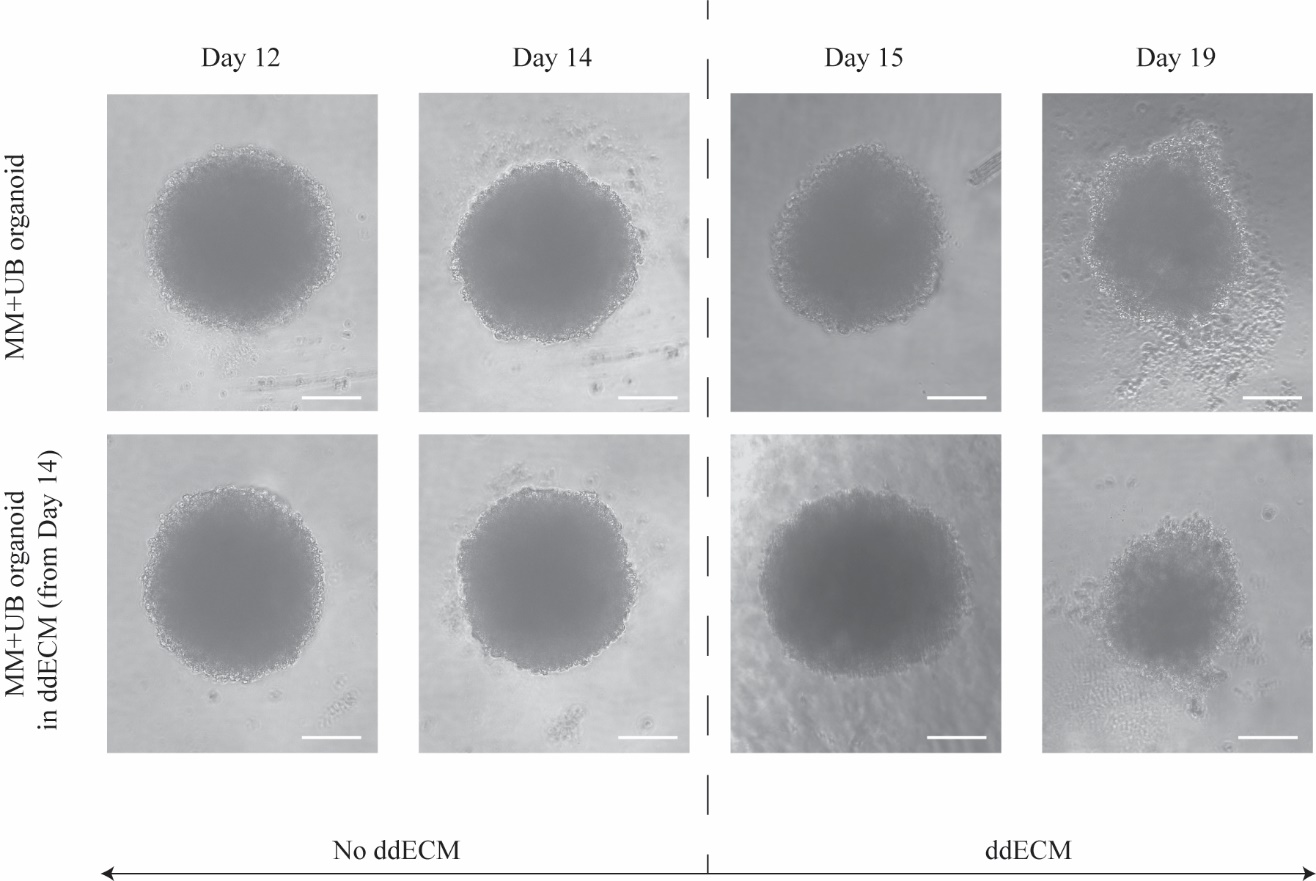


Figure S2: **Representative brightfield images of MM+UB organoids from Day 12 to Day 19, investigating ddECM embedding.** On Day 14 the differentiation was over (Figure 1a), and organoids were cultured in Adv RPMI supplemented with 1% v/v GlutaMax, with no GF, testing the embedding in ddECM. Images were taken with microscope CKX53 Olympus. Scale bar: 100 µm.


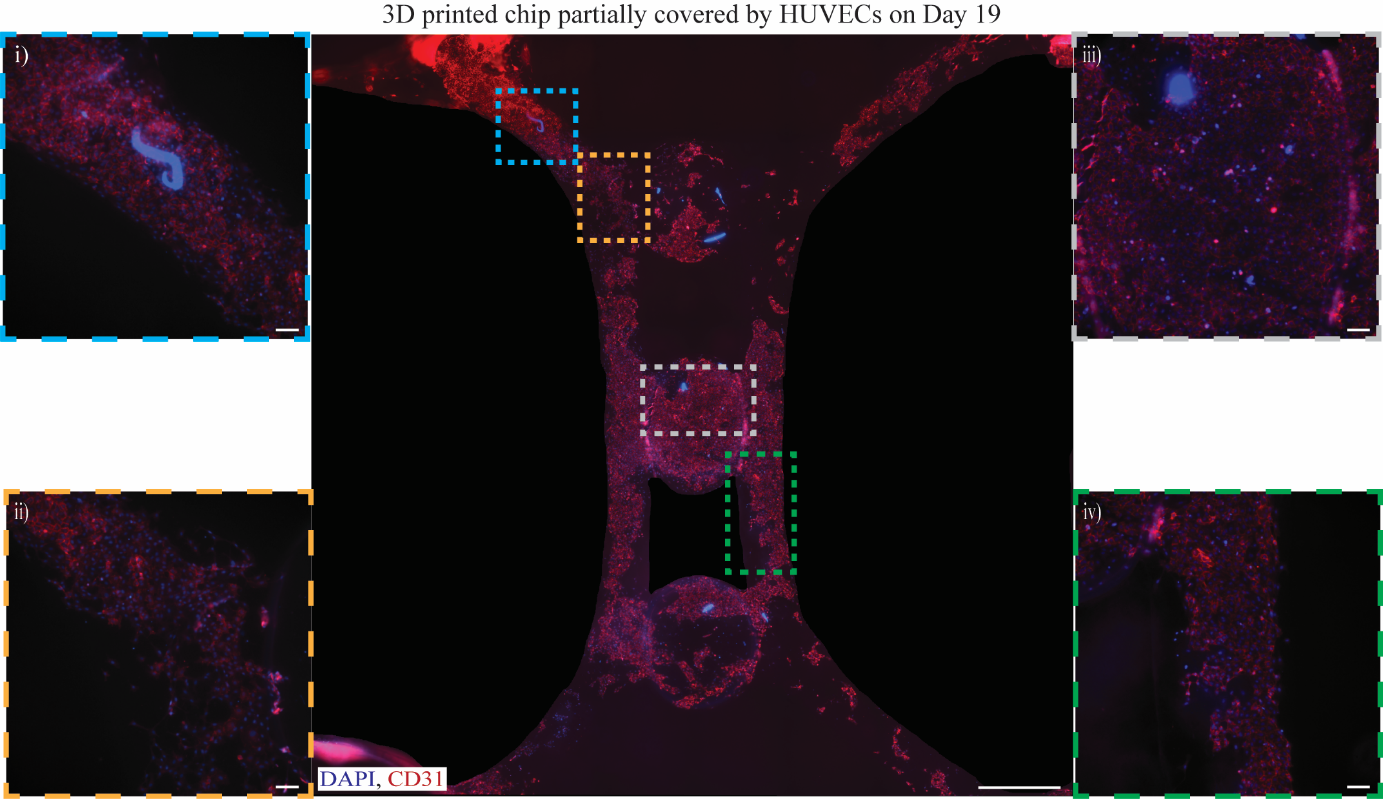


Figure S3: **HUVECs cultured in 3D printed chip, showing partially detachment on Day 19.** HUVECs were seeded in the 3D printed chip on Day 7, and cultured until Day 14 in their native medium EGM-2. On Day 14, the medium was changed to Adv RPMI, and the HUVECs were cultured until Day 19, showing a detachment over time, however still expressing an endothelial phenotype, as highlighted by the zoom in images i-iv). Scale bar: 1000 µm for overall chip, and for zoom in images i-iv) 100 µm. DAPI staining is shown in blue and CD31 in red.

Table S1: **List of proteins in the acellular ddECM hydrogel formulation.** Proteins’ intensities were quantified in the ddECM hydrogel formulation after digestion overnight with pepsin. Proteins listed in alphabetic order.

*
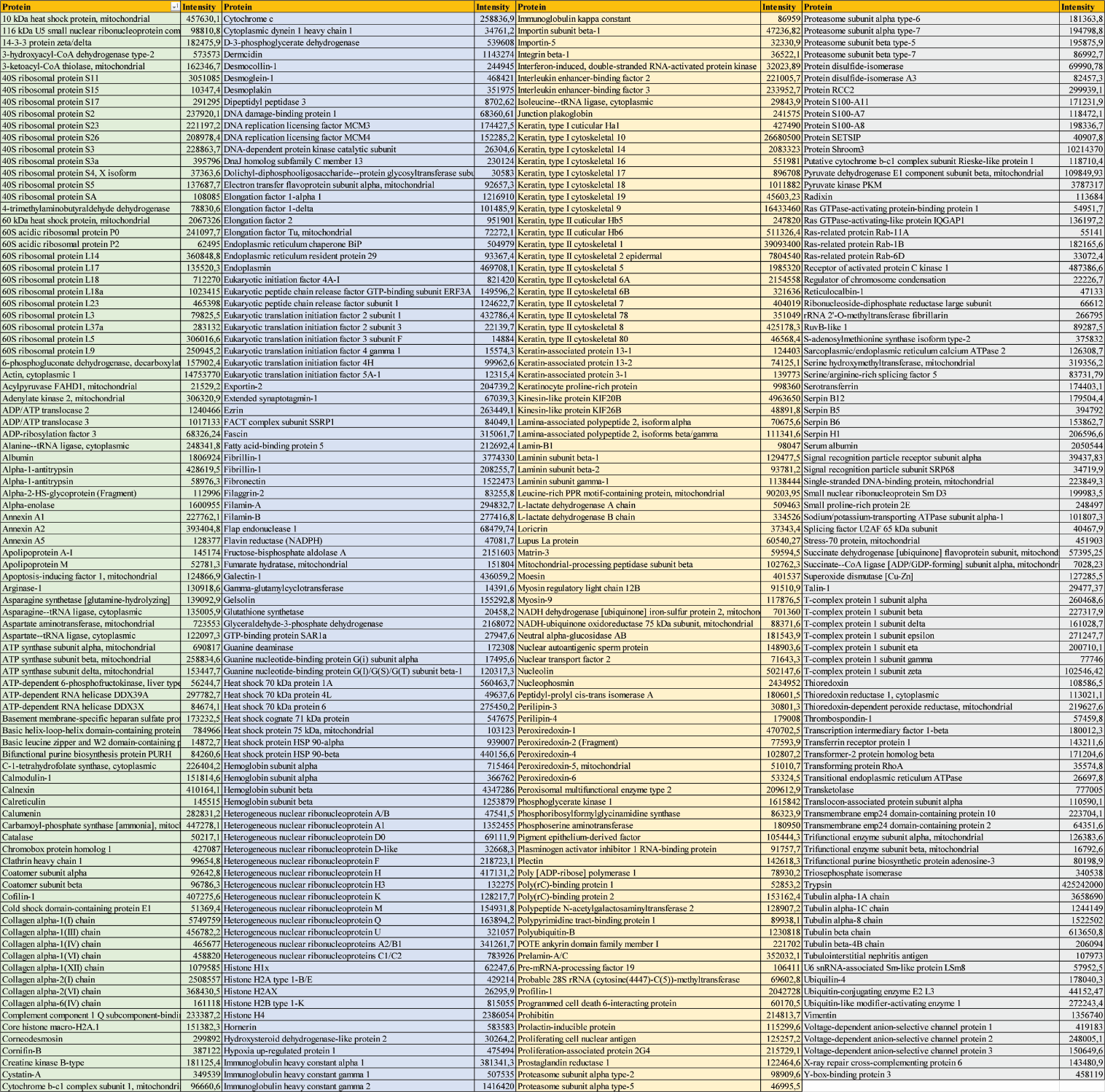
*

Video S1: **Z-stack of the immunostained 3D ddECM gel casted above a monolayer of HUVECs in 2D**, DAPI is stained in blue, and CD31 in red. Scale bar: 100 µm.

Video S2: **Z-stack of the brightfield 3D ddECM gel casted above a monolayer of HUVECs in 2D**. Scale bar: 100 µm.

Video S3: **Z-stack of the central part of the organoid, showing the branching.** DAPI is stained in blue, and CD31 in red. Scale bar: 100 µm.

Video S4: **Z-stack of the outer side of the organoid, showing the branching.** DAPI is stained in blue, and CD31 in red. Scale bar: 100 µm.

Video S5: **Z-stack of the vascularized top gel compartment.** Here it is possible to see the interaction between the glomerulus-like areas stained by PODXL stained in yellow and capillary-like areas stained by CD31 in red, plus the nuclei stained in blue. Scale bar: 100 µm.

Video S6: **Z-stack of the vascularized central gel compartment.** Here it is possible to see the interaction between the glomerulus-like areas stained by PODXL stained in yellow and capillary-like areas stained by CD31 in red, plus the nuclei stained in blue. Scale bar: 100 µm.

Video S7: **Z-stack of the vascularized bottom gel compartment.** Here it is possible to see the interaction between the glomerulus-like areas stained by PODXL stained in yellow and capillary-like areas stained by CD31 in red, plus the nuclei stained in blue. Scale bar: 100 µm.
